# Impact of CT Injector Technology and Contrast Media Viscosity on Vascular Enhancement: Evaluation in a Circulation Phantom

**DOI:** 10.1259/bjr.20190868

**Published:** 2020-04-13

**Authors:** Michael McDermott, Corey Kemper, William Barone, Gregor Jost, Jan Endrikat

**Affiliations:** 1 Bayer U.S. LLC, Bayer Pharmaceuticals, Radiology R&D, Indianola, PA 15051, USA; 2 Bayer AG, MR & CT Contrast Media Research, Berlin, Germany; 3 Bayer AG, Radiology R&D, 13353 Berlin, Germany; 4 Department of Gynecology, Obstetrics and Reproductive Medicine, University Medical School of Saarland, 66421 Homburg/Saar, Germany

## Abstract

**Objective:**

To assess the impact of piston-based *vs* peristaltic injection system technology and contrast media viscosity on achievable iodine delivery rates (IDRs) and vascular enhancement in a pre-clinical study.

**Methods:**

Four injectors were tested: MEDRAD^®^ Centargo, MEDRAD^®^ Stellant, CT Exprès^®^, and CT motion^™^ using five contrast media [iopromide (300 and 370 mgI ml^−1^), iodixanol 320 mgI ml^−1^, iohexol 350 mgI ml^−1^, iomeprol 400 mgI ml^−1^]. Three experiments were performed evaluating achievable IDR and corresponding enhancement in a circulation phantom.

**Results:**

Experiment I: Centargo provided the highest achievable IDRs with all tested contrast media (*p* < 0.05). Iopromide 370 yielded the highest IDR with an 18G catheter (3.15 gI/s); iopromide 300 yielded the highest IDR with 20G (2.70 gI/s) and 22G (1.65 gI/s) catheters (*p* < 0.05).

Experiment II: with higher achievable IDRs, piston-based injectors provided significantly higher peak vascular enhancement (up to 48% increase) than the peristaltic injectors with programmed IDRs from 1.8 to 2.4 gI/s (*p* < 0.05).

Experiment III: with programmed IDRs (*e.g.* 1.5 gI/s) achievable by all injection systems, Centargo, with sharper measured bolus shape, provided significant increases in enhancement of 34–73 HU in the pulmonary artery with iopromide 370 (*p* < 0.05).

**Conclusion:**

The tested piston-based injection systems combined with low viscosity contrast media provide higher achievable IDRs and higher peak vascular enhancement than the tested peristaltic-based injectors. With equivalent IDRs, Centargo provides higher peak vascular enhancement due to improved bolus shape.

**Advances in knowledge:**

This paper introduces a new parameter to compare expected performance among contrast media: the concentration/viscosity ratio. Additionally, it demonstrates previously unexplored impacts of bolus shape on vascular enhancement.

## Introduction

The practice of CT intravenous contrast media (CM) administration has been continuously debated and updated^1^; however, performance of power injection systems has largely gone unstudied. Previous publications have focused on workflow efficiency as the main differentiator between systems.^2,3^ Further, patient-to-patient variability, as well as inconsistencies in scanner protocols and technology can mask differences in the technical capabilities of the injection systems.

When looking at differences between injection systems, there are two main fluid delivery technologies to analyze: piston-based systems and peristaltic-pump-based systems. Both are positive displacement pumps, which translate kinetic energy from the pump into displacement of a given quantity of fluid. However, piston-based systems act by using a piston/ram to displace a plunger down the barrel of a reservoir, operating in two directions to first fill the reservoir and then to displace the fluid from the reservoir to the patient, similar to a hand syringe. Peristaltic pumps act as rotary pumps, using rollers to pinch sections of flexible tubing which draws fluid directly from the supply source and displaces it out to the patient. These differences in fluid delivery technology can cause differences in performance. In early 2019, Chaya et al^4^ published results of a pre-clinical study in which a piston-based injection system demonstrated higher maximum achievable flow rates and more consistent steady-state flow when compared to the two tested peristaltic pump-based injection systems.^4^ The authors, however, acknowledged several limitations of their study. Primarily, the study included only two CM types spanning a small range of viscosities, a critical element when determining the iodine delivery rates (IDRs) achievable for a given injection system. This is because increased contrast viscosity results in increasing injection pressures.^5^ An additional limitation of Chaya et al was that no link was provided between measured performance and potential clinical outcomes.

Building upon the work of Chaya et al, this study aimed to evaluate the technical performance of contemporary power injection systems by applying a broader range of CM concentrations and viscosities, as well as more sophisticated measurement techniques to determine flow rates and injected concentrations. In addition, a circulation phantom was used to quantify vascular enhancement in CT while minimizing experimental variables. Similar phantoms have been used in previous experiments to study the impact on CT imaging of variables such as different catheter types,^6^ CM viscosity/temperature,^5^ saline use,^7^ contrast injection protocols for low kVp imaging,^8^ and iodine delivery rate.^9^

In the present study, four injection systems and five CM were compared across a range of catheter gauges. Three experiments were performed to determine: (I) contribution of injection system technology and CM viscosity to maximum achievable IDR; (II) impact of differences in achievable IDR on vascular enhancement in a circulation phantom; and (III) impact of injection system technology and bolus shape on peak enhancement with the same achievable IDRs.

## Methods

### Injection Systems and Contrast Media

Four CT injection systems were used from three manufacturers: MEDRAD^®^ Centargo CT Injection System and MEDRAD^®^ Stellant CT Injection System with the Multi Patient Kit (‘Centargo’ and ‘Stellant MP’, Bayer AG, Berlin, Germany), Bracco CT Exprès^®^ Contrast Injection System with Multi Patient Set (‘CT Exprès’, Bracco Injeneering, Lausanne, Switzerland) and ulrich CT motion™ Contrast Media Injector (‘CT motion’, ulrich Medical, Ulm, Germany). All injection systems were operated according to the manufacturers’ instructions with disposable components designed for multiple injections/patients, with an exception for CT Exprès, which requires selection of the catheter gauge to restrict the programmable flow rate of the injection system. As the flow rate restrictions prevent programming of equivalent IDRs to the other tested injection systems, the catheter gauge was intentionally selected as a lower gauge than that which was used. The systems were connected to 18G, 20G, and 22G intravenous catheters (Becton Dickinson, Franklin Lakes, NJ) to generate a range of expected clinical injection pressures.^10^ Details on the tested CM are shown in Table 1. All CM tested were at room temperature, which was monitored during the study to be at 21.5°C.

**Table 1.** Contrast media used for this study, with properties reported by the manufacturer, measured viscosity, and the derived concentration/viscosity ratio

Generic (Brand Name)	Concentration (mgI/mL)	Published Viscosity (cP)(^a^)	Measured Viscosity (cP)(^b^)	Concentration / Viscosity Ratio (mgI/mL/cP)(^c^)	Concentration / Viscosity Ratio (mgI/mL/cP) at 37 ºC(^d^)	Manufacturer
Iopromide (Ultravist)^11^	300	9.2	7.64	39.3	61.2	Bayer AG Berlin, Germany
Iodixanol (Visipaque)^12^	320	26.6	21.1	15.2	27.1	GE Healthcare New Jersey, USA
Iohexol (Omnipaque)^13^	350	20.4	18.7	18.7	33.7	GE Healthcare New Jersey, USA
Iopromide (Ultravist)^14^	370	22	17.1	21.6	37	Bayer AG Berlin, Germany
Iomeprol (Iomeron)^15^	400	27.5	23	17.4	31.7	Bracco Imaging Milan, Italy

a Official data from manufacturers at 20°C.

b Measured data using Brookfield DV-II+ Pro Viscometer at tested temperature of 21.5°C.

c Determined using measured contrast media viscosity.

d Calculated from manufacturer reported viscosities at 37°C.

### Real-time Density and Volumetric Flow Rate Measurement

Real-time density and volumetric flow rate were measured using a MicroMotion 5700 Coriolis Transmitter (Emerson Electric Co., St. Louis, MO). The transmitter was positioned at the outlet of the catheter to record the bolus shape as measured by the concentration and flow rate of the fluid exiting the catheter over time. The signal from the transmitter was recorded using a LabVIEW virtual instrument (2012 SPI, National Instruments). This measurement method allows for actual IDRs to be calculated, irrespective of the programmed IDR for that injection.

### Circulation Phantom

**Figure 1.**
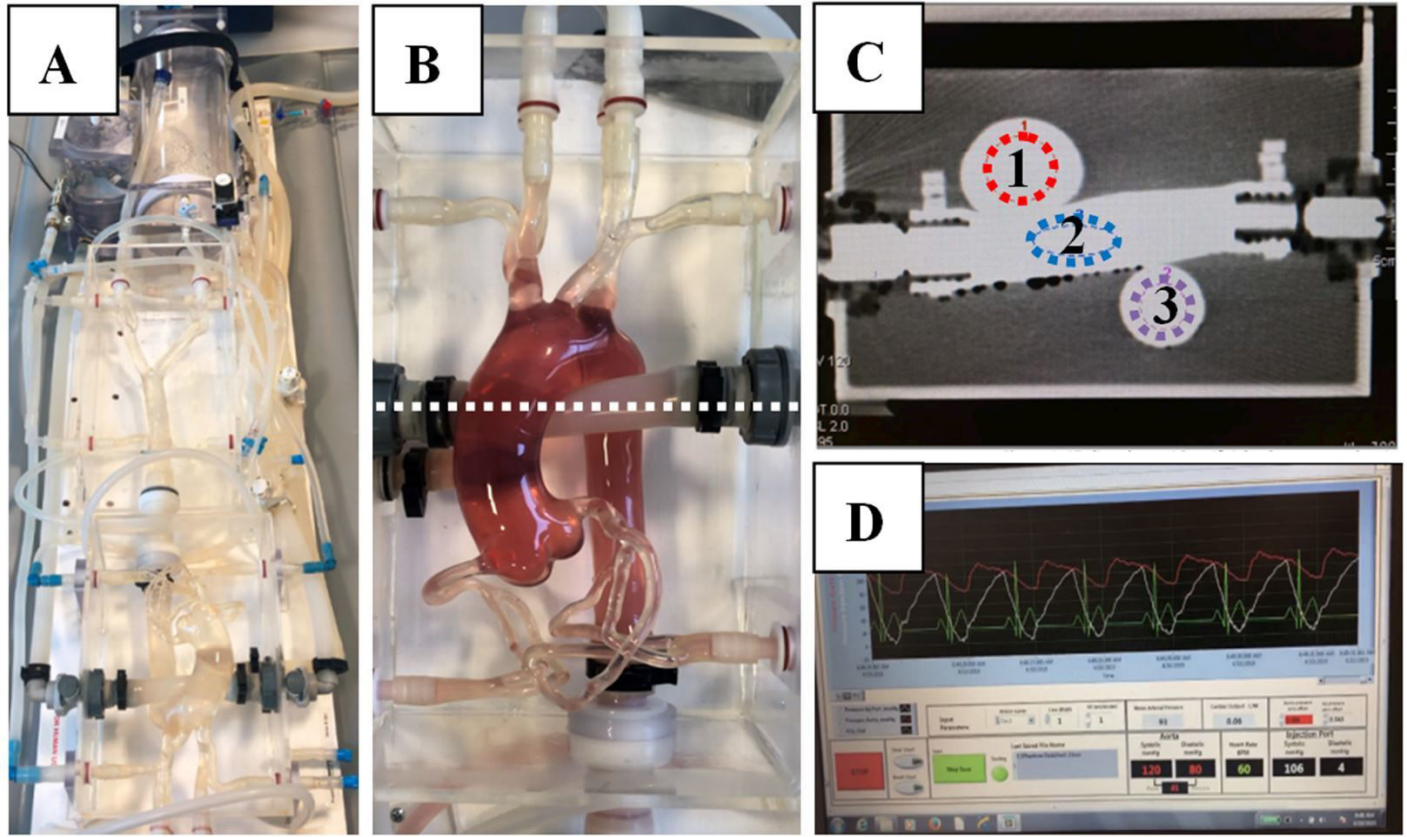
Circulation phantom A: Circulation phantom; B: Aortic arch + pulmonary artery with dyed fluid to enhance visibility of vasculature. Dye was not used for experimental trials; C: Cross-section through B (dotted line) showing regions of interest: 1 = ascending aorta, 2 = pulmonary artery, 3 = descending aorta, D: Graphical monitor of blood pressure and heart rate of the phantom.

A circulation phantom was used to assess the effects of maximum achievable IDR and bolus shape on vascular enhancement in CT. The phantom mimics the transport and distribution dynamics of iodinated contrast material in a mammalian circulatory system. The design of the phantom is equivalent to those used in previously published experiments.^6–8^ For all acquisitions, the phantom was configured to have a heart rate of 60 beats per minute and a blood pressure of 120/80 mmHg (Figure 1). The phantom was drained and re-filled with laboratory water between each injection to prevent the effects of residual contrast material contamination across injections.

### CT Scanner Settings & Image Evaluation

All CT acquisitions were performed on a dual-source CT scanner (SOMATOM Force; Siemens Healthineers, Erlangen, Germany) with a tube voltage of 120 kV and tube current of 100 mAs. A dynamic CT of the aortic arch was acquired without table feed utilizing the following parameters: 200 mm field of view, 2.0 mm slice thickness, 40 s scan duration, and 0.5 s rotation time. Image reconstruction was performed with a temporal resolution of 0.3 s enabling the monitoring of the bolus curve. Regions of interest (ROIs) were drawn at the level of the ascending aorta (AA), descending aorta (DA), as well as within the pulmonary artery (PA), as shown in Figure 1c. Equivalent enhancement trends and magnitudes were observed in the AA and DA; therefore, only the results for the AA are included for simplicity. The peak signal enhancement was derived from the resulting time-enhancement curve. All CT scans were repeated three times and measurements taken within the same slice plane to ensure accuracy, determine repeatability, and allow for statistical analysis across groups. Methods to obtain enhancement values are consistent with previously published results.^8^

### Statistical Analysis

Independent non-parametric Kruskal–Wallis tests using α = 5% as a significance level were conducted for comparison of peak enhancement, IDR, contrast media, and injection system. Each analysis was conducted independently across each ROI and catheter gauge. As an exploratory analysis, *p*-values ≤ 0.05 were interpreted to be statistically significant. Minitab statistical software (v. 17, Minitab, LLC, State College, PA) was used for all analyses.

### Experiment I—Maximum Achievable IDR

Maximum achievable IDRs were determined for all four injection systems delivering five CM through 18G, 20G, and 22G catheters. Each of these three variables was varied to generate a testing matrix of 60 combinations. Injections were programmed at 1 ml s^−1^ and increased in 0.5 ml s^−1^ increments until the injection systems were unable to deliver the flow rate without exceeding the maximum programmable pressure limit as selected on or defined by the system. Injection volumes of 100 ml were chosen to ensure steady state flow was achieved.

### Experiment II—Relationship Between IDR and Peak Enhancement

In order to relate the differences in achievable IDR to potential clinical results, dynamic CT acquisitions were obtained using the phantom. The impact of achievable IDR on peak vascular enhancement was assessed with specific focus on the contribution of the injection systems. This was achieved by varying the programmed IDR from 1.8 to 2.4 gI/s in 0.2 gI/s increments. Iopromide 300 and 370 were used in this experiment, as they achieved the highest IDRs in Experiment I for all injection systems. This allowed for comparison of all injection systems at the higher end of clinically relevant IDRs. Volumes were varied to maintain constant duration across the programmed injections.

### Experiment III—Impact of Bolus Geometry on CT Peak Vascular Enhancement

Experiment III was designed to compare differences in peak vascular enhancement with common injection protocols. Specifically, IDRs of 1.5 and 2.0 gI/s were utilized in this study, consistent with literature for adequate vascular enhancement in CT angiography procedures.^16–18^ By comparing vascular enhancement at IDRs that are achievable by all systems, differences can be attributed to injection system performance characteristics such as bolus shape.

To maintain consistency with Experiment II, iopromide 300 and 370 were used. In order to achieve an IDR of 1.5 gI/s, all injectors were programmed to deliver at 5 and 4.1 ml s^−1^ for iopromide 300 and iopromide 370, respectively. Similarly, to achieve an IDR of 2.0 gI/s, all injectors were programmed to deliver at 6.7 and 5.4 ml s^−1^ for iopromide 300 and iopromide 370, respectively. The volumes (40 and 54 ml) were appropriate relative to the central blood volume of the phantom and ensured the injection reached steady state, while avoiding recirculation effects.

## Results

### Experiment I—Maximum Achievable IDR (Figure 2, Table 2)

**Figure 2.**
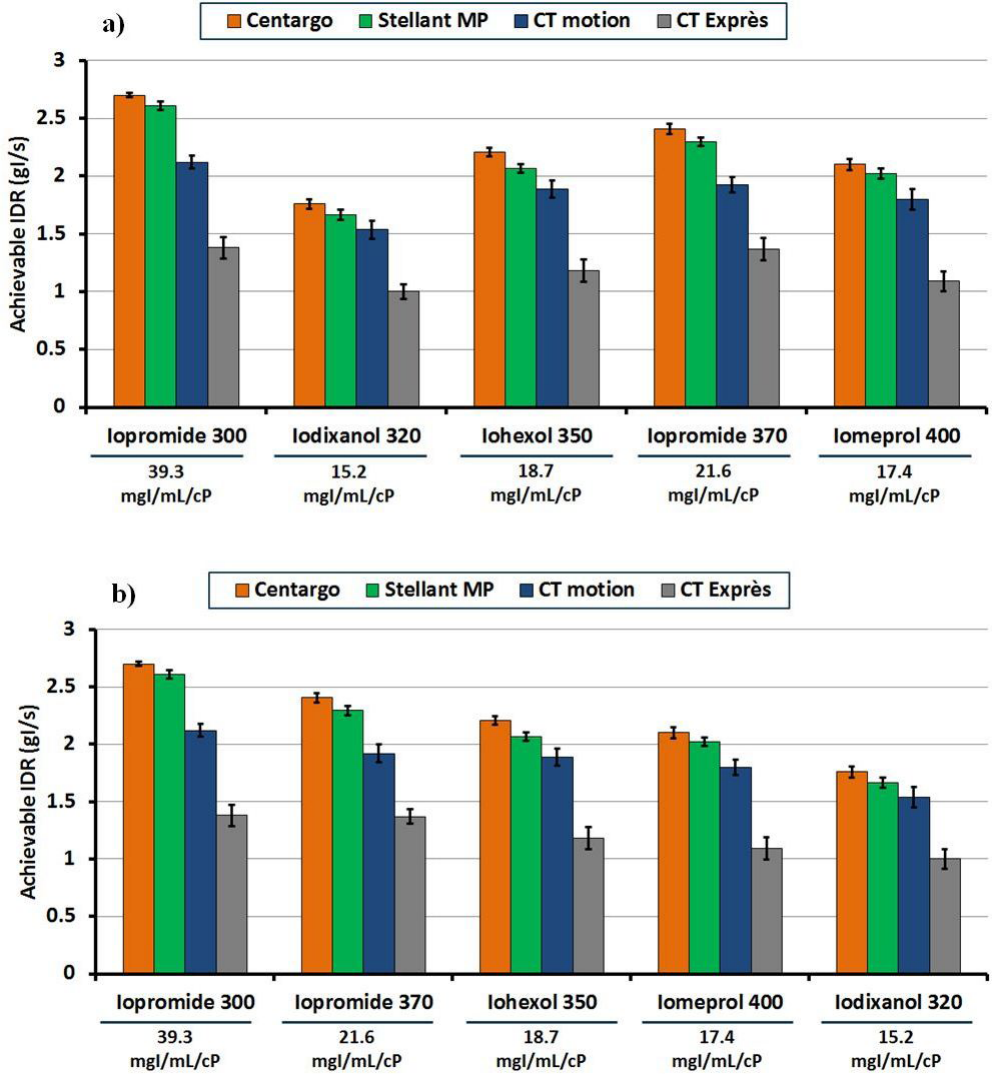
Maximum achievable IDR vs injection system with different contrast media through a 20G catheter: This figure represents the maximum achievable IDR for each combination of injection system and contrast media through a 20G catheter. The measured concentration/viscosity ratio is shown below each contrast media in mgI/mL/cP. Error bars represent standard deviation across the three trials performed for each programmed injection. Graph A represents the data ordered from left to right by concentration, while graph B represents the data ordered from left to right by measured concentration/viscosity ratio. IDR,iodine delivery rate.

**Table 2.** Complete results for maximum achievable IDR vs injection system vs contrast media vs catheter gauge: values represent the average of three trials for each combination of contrast media, injection system, and catheter gauge

Catheter gauge	Contrast media	Concentration (mgI/mL)	Maximum iodine delivery rate (gI/s)			
			Centargo	Stellant MP	CT motion	CT Exprès
18G	Iopromide	300	3.00	3.00	2.70	1.59
	Iodixanol	320	2.27	2.18	2.02	1.18
	Iohexol	350	2.91	2.77	2.49	1.40
	Iopromide	370	3.15	3.07	2.70	1.63
	Iomeprol	400	2.48	2.40	2.14	1.32
20G	Iopromide	300	2.70	2.61	2.12	1.38
	Iodixanol	320	1.76	1.66	1.54	1.00
	Iohexol	350	2.21	2.07	1.89	1.18
	Iopromide	370	2.41	2.29	1.92	1.37
	Iomeprol	400	2.10	2.02	1.80	1.09
22G	Iopromide	300	1.65	1.56	1.26	0.99
	Iodixanol	320	1.12	1.02	0.78	0.66
	Iohexol	350	1.37	1.16	0.98	0.81
	Iopromide	370	1.48	1.41	1.13	0.83
	Iomeprol	400	1.28	1.16	0.88	0.74

IDR, iodine delivery rate.

Centargo provided the highest achievable IDRs among the tested injection systems with all five CM and all catheter gauges (*p* < 0.05) (Table 2). Iopromide 370 yielded the highest IDR among the tested CM with an 18G catheter (3.15 gI/s) (*p* < 0.05), while iopromide 300 yielded the highest IDR with both a 20G (2.70 gI/s) and 22G (1.65 gI/s) catheter (*p* < 0.05). Following iopromide across all catheter gauges were iohexol 350, iomeprol 400, and then iodixanol 320. With a 20G catheter, Centargo with iopromide 300 achieved the highest IDR (2.7 gI/s), while CT Exprès with iodixanol 320 achieved the lowest IDR (1.0 gI/s) (Figure 2).

### Experiment II—Relationship Between IDR and Peak Vascular Enhancement (Figure 3)

**Figure 3.**
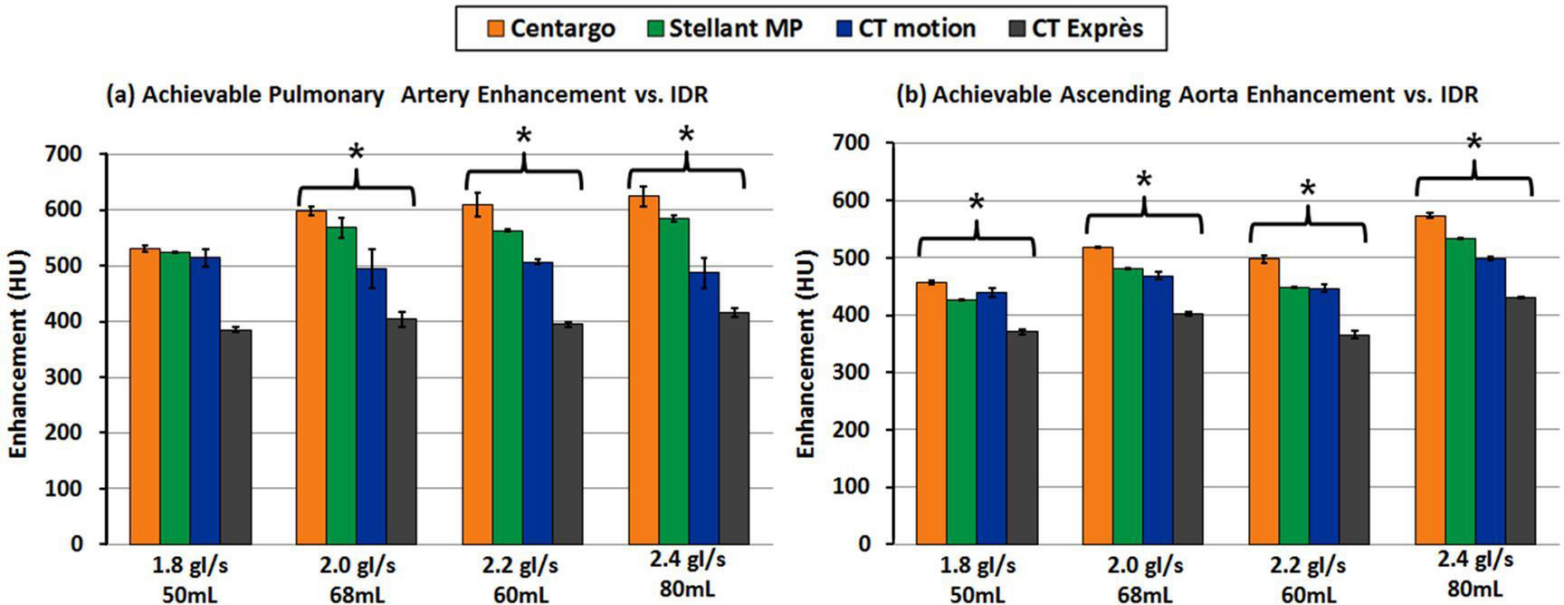
Achievable vascular enhancement *vs* injection system with varied IDRs through a 22G catheter: The left graph (a) provides peak enhancement results from all injection systems in the pulmonary artery, while the right graph (b) was acquired from the ascending aorta. Error bars represent standard deviation across the three trials performed for each programmed injection. * denotes statistical difference between injection systems (*p* < 0.05).

In all measured ROIs, Centargo provided the highest peak vascular enhancement of the tested injection systems across all programmed IDRs ranging from 1.8 to 2.4gI/s. Statistically significant results (*p* < 0.05) were recorded for all tested IDRs, except for the pulmonary artery at 1.8 gI/s (Figure 3). With a programmed IDR of 2.0 gI/s, Centargo achieved an average peak enhancement increase of 104–195 Hounsfield units (HUs) in the pulmonary artery, and 50–116 HU in the ascending aorta compared to both peristaltic injection systems. Enhancement results across all programmed IDRs are shown in Table 3.

**Table 3.** Mean increase in vascular enhancement across all tests of Figure 3 of Centargo vs other injectors: the table represents the mean increase in vascular enhancement across all injections with programmed IDRs from 1.8 to 2.4 gI/s

		Enhancement in pulmonary artery			Enhancement in ascending aorta		
Programmed IDR (gI/s)	Injection system	Mean (HU)	Variation (HU)	% Difference of means (relative to Centargo)	Mean (HU)	Range (HU)	% Difference of means (relative to Centargo)
1.8–2.4 gI/s	Centargo	591	531–624	-	512	457–574	-
	Stellant MP	560	524–584	−6%	473	427–534	−8%
	CT motion	500	494–514	−18%	463	440–499	−11%
	CT Exprès	400	385–416	−48%	392	366–430	−31%

HU, Hounsfield unit; IDR, iodine delivery rate.

Iopromide 300 and iopromide 370 were used with a 22G catheter. Differences in HU are represented as a percentage relative to Centargo as the top performing injection system.

### Experiment III—Bolus Geometry Impacts Peak Vascular Enhancement (Figure 4)

**Figure 4.**
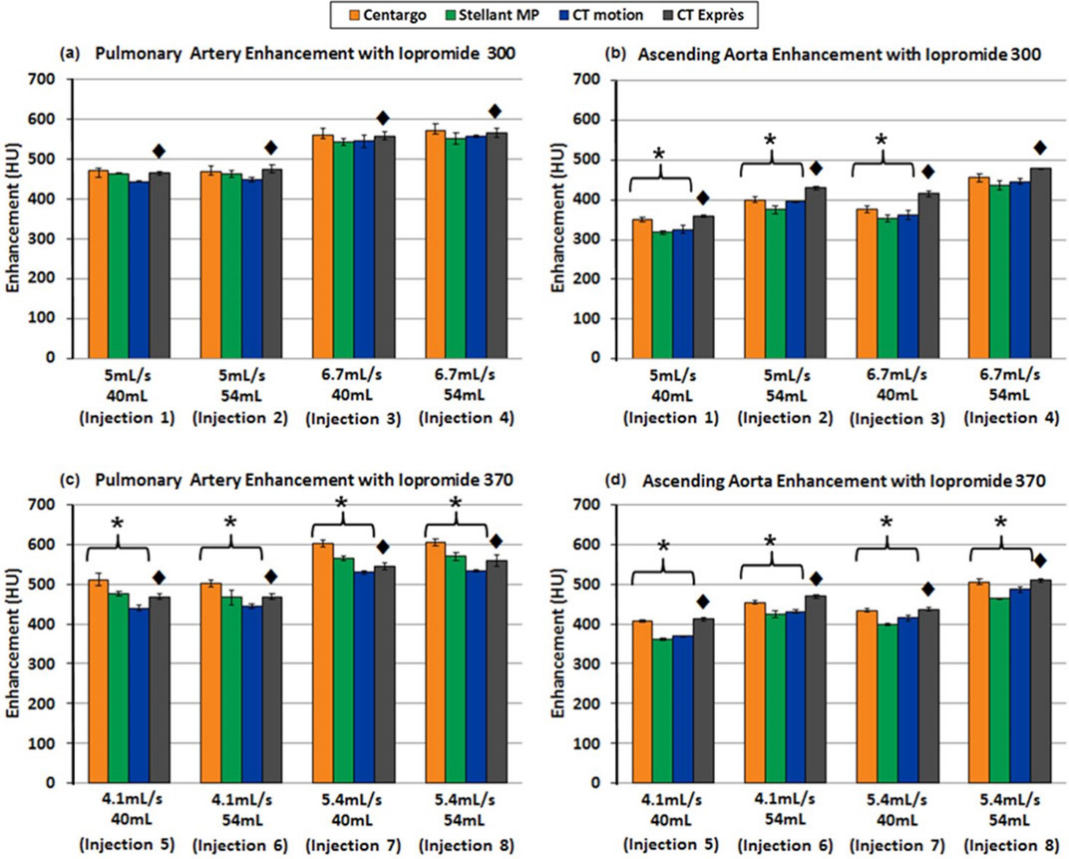
Achievable vascular enhancement vs injection system with varied IDRs through a 20G catheter: the top two graphs (a, b) provide results using iopromide 300, while the bottom graphs (c, d) utilized iopromide 370. In addition, (a, c) are measurements from the pulmonary artery while (b, d) are measurements from the ascending aorta. Error bars represent standard deviation across the three trials performed for each programmed injection. ♦ denotes data obtained using the CT Exprès injection system where iodine was over delivered. Due to the additional contrast media, comparisons with CT Exprès were not considered, though the data are provided for completeness. * denotes statistical difference between injection systems (*p* < 0.05).

In general, Centargo and CT Exprès provided greater enhancement, however CT Exprès was excluded from statistical analysis due to observed over delivery of contrast (see "Discussion"). With iopromide 300, Centargo provided an average increase of ~14–26 HU and ~5–32 HU in the PA and AA respectively. Centargo demonstrated significantly increased enhancement in the PA with iopromide 370, with a 34–73 HU increase relative to the other tested injection systems (Figure 4c). Similar differences in peak enhancement were observed in the AA with iopromide 370 (*p* < 0.05).

## Discussion

This study assessed the impact of injection system technologies and CM viscosity on achievable iodine delivery rates and vascular enhancement. In order to translate IDR to vascular enhancement, a circulation phantom was used in combination with dynamic CT. This provided a stable model that, unlike patients in a clinical setting, could be injected into and scanned many times under standardized conditions.

### Experiment I—Maximum Achievable IDR

Consistent with Chaya et al,^4^ piston-based systems, Centargo and Stellant MP, had higher achievable flow rates than peristaltic-based systems, CT motion and CT Exprès across all CM tested. The combination of the fluid delivery technology and differences in maximum programmable pressure limits were the key contributors to observed differences in achievable flow rates and corresponding IDRs.

Across CM types, the higher viscosity agents (iodixanol 320 and iomeprol 400) had the lowest achievable IDRs for all injection systems. This is not necessarily an intuitive result, as theoretical maximum IDR increases with increasing concentration.^18,19^ This study has found the impact of increasing viscosity is greater than the effect of increasing concentration. Still, a balance of both properties is important for determining achievable IDR and achievable vascular enhancement. We propose a new parameter for CM performance by calculating the ratio of concentration to viscosity (Table 1). Figure 2b demonstrates a strong correlation of decreased achievable IDR with decreasing concentration/viscosity ratio (R^2^ >0.92 for all combinations of catheter gauge and injection system). While not tested in this study, Table 1 includes the calculated concentration/viscosity ratios for all tested CM if warmed to 37°C. It is expected that achievable IDRs increase for all contrast media at higher temperatures, however, differences in viscosity with heated CM are still expected to lead to differences in performance.

Yet, based on manufacturer reported viscosity, the concentration/viscosity ratio was not able to explain the improved maximum achievable IDR of iopromide 370 relative to iohexol 350. Upon review of the manufacturer’s data, published CM viscosities were obtained at a temperature of 20^°^C. However, using a calibrated viscometer (Brookfield DV-II +Pro, AMETEK Inc.) the viscosity of iohexol 350 at the ambient testing laboratory temperature of 21.5^°^C was measured to be higher than that of iopromide 370 (18.70 and 17.10 cP respectively). Correcting the concentration/viscosity ratio for the temperature of the testing environment explains the improved performance for iopromide 370 (Table 1). This result aligns with previous observations relating achievable IDR and concentration/viscosity ratio while highlighting the dependence of CM viscosity on temperature.

Iopromide 300, with the highest measured concentration/viscosity ratio of 39.3 mgI ml^−1^/cP, produced the highest achievable IDRs using a 20G and 22G catheter by a significant margin (*p* < 0.05). With an 18G catheter, iopromide 370 produced the highest achievable IDR (3.15 gI/s), representing the only result where the highest measured concentration/viscosity ratio CM did not achieve the highest IDR. This is explained as iopromide 300 was restricted to 3.0 gI/s due to a maximum programmable flow rate of 10 ml s^−1^ on the tested injection systems, a limitation of the systems and not the CM. Conversely, iodixanol 320 has the lowest measured concentration/viscosity ratio of 15.2 mgI ml^−1^/cP. As expected, this resulted in the lowest achievable IDRs among the tested CM for all catheter gauges.

### Experiment II—Relationship Between IDR and Peak Enhancement

While the data from Experiment I established differences between injection systems and CM for achievable IDR, Experiment II provided potential clinical relevance for these results.

**Figure 5.**
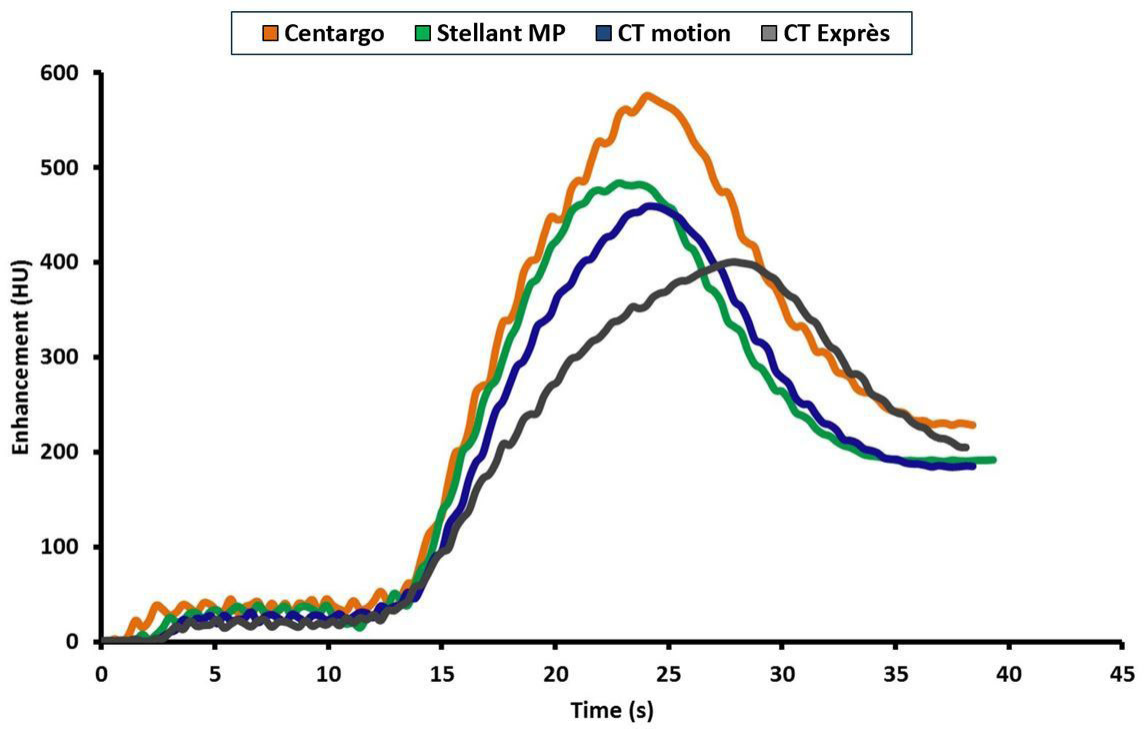
Sample aortic enhancement comparison of injection systems with 2.0 gI/s programmed injection (time shifted based on contrast media arrival time): This figure represents the enhancement profile in the ascending aorta of the phantom when the same 2.0 gI/s injection is executed on each of the four injection systems. The impact of reduced achievable IDRs as the systems reduce flow to limit pressure is seen in the enhancement profiles and resulting peak enhancement values.

Before analyzing the results, it is important to note the difference between programmed IDRs and achievable IDRs. While programmed IDR is theoretical, based on the programmed rate and concentration used, achievable IDR is the measured flow rate and concentration exiting the catheter. Differences between programmed and achieved IDR arise from injection system performance. For example, when the pressure limit of the injection system is reached, the systems reduce the flow rate to prevent the pressure from exceeding the maximum limit. This reduction in flow rate decreases the achieved IDR, which is shown to decrease peak vascular enhancement (Figure 5).

In the PA and AA of the phantom, Centargo provided the highest peak vascular enhancement of all four injection systems across the programmed IDRs (Table 3). These results can be explained by the inability of the peristaltic injection systems to reach the programmed IDR, as the achieved IDR for CT motion and CT Exprès were markedly lower. This is attributed to a reduction in flow rate by both systems to prevent pressure from exceeding the maximum limit, as previously discussed. Review of the data from Experiment II (Figure 5) shows the clinical impact of this flow reduction, which is seen as broadening of the CM bolus and subsequent enhancement profile. The resulting enhancement is reduced and the peak is shifted later in time. This analysis further illustrates the relationship between achievable IDR and peak vascular enhancement.

### Experiment III—Bolus Shape Impacts Peak Vascular enhancement

**Figure 6.**
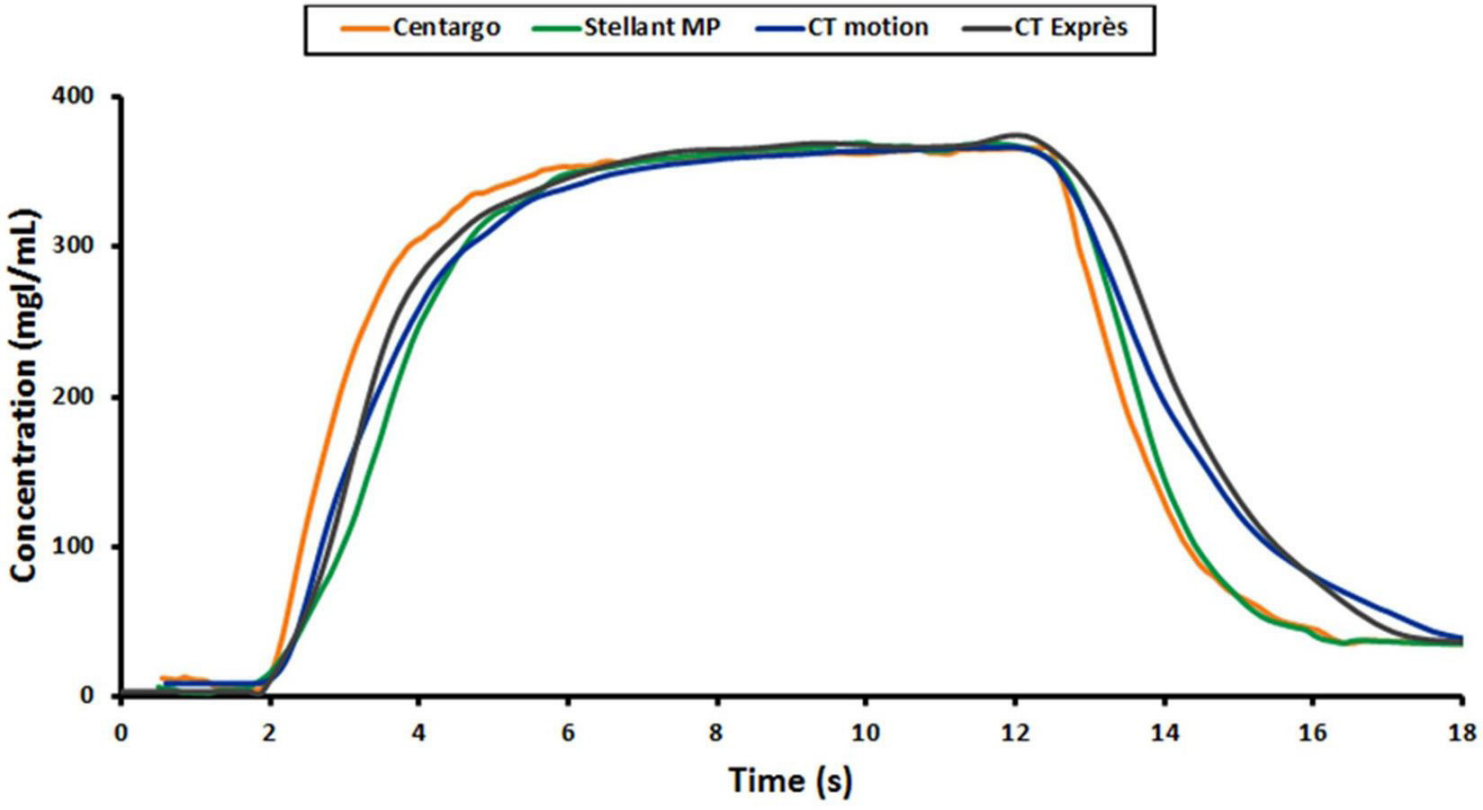
Sample of contrast bolus shape comparison between injection systems: this figure represents the concentration plots as measured at the 20G catheter for Injection 8 of Experiment III. Differences in leading and trailing edge of the bolus can be seen between injection systems.

While the previous experiment established the performance differences between the injection systems with IDRs at the higher end of the clinically relevant range, Experiment III compared the injection systems using nominal protocols for CT angiography with the most common catheter gauge. As the experiment was designed to ensure all injection systems were able to achieve the programmed IDRs without reaching their maximum pressure limit, the resulting vascular enhancement should be dependent only on bolus shape. Concentration curves generated using the Coriolis Transmitter allow for comparison of bolus shapes across injection systems (Figure 6).

Theoretically, optimal bolus shape (not enhancement profile) is recognized as a square wave, with concentration of the fluid entering the patient rising instantaneously to the desired level at the start of the injection and falling instantaneously with the onset of the saline flush. As seen in Figure 6, Centargo was found to provide sharper leading and trailing edges of the bolus. This improved bolus shape provides more efficient use of CM, with more iodine entering the patient at the desired IDR for a longer duration. These differences in bolus shape largely explain the general increase in enhancement provided by Centargo in Experiment III.

**Figure 7.**
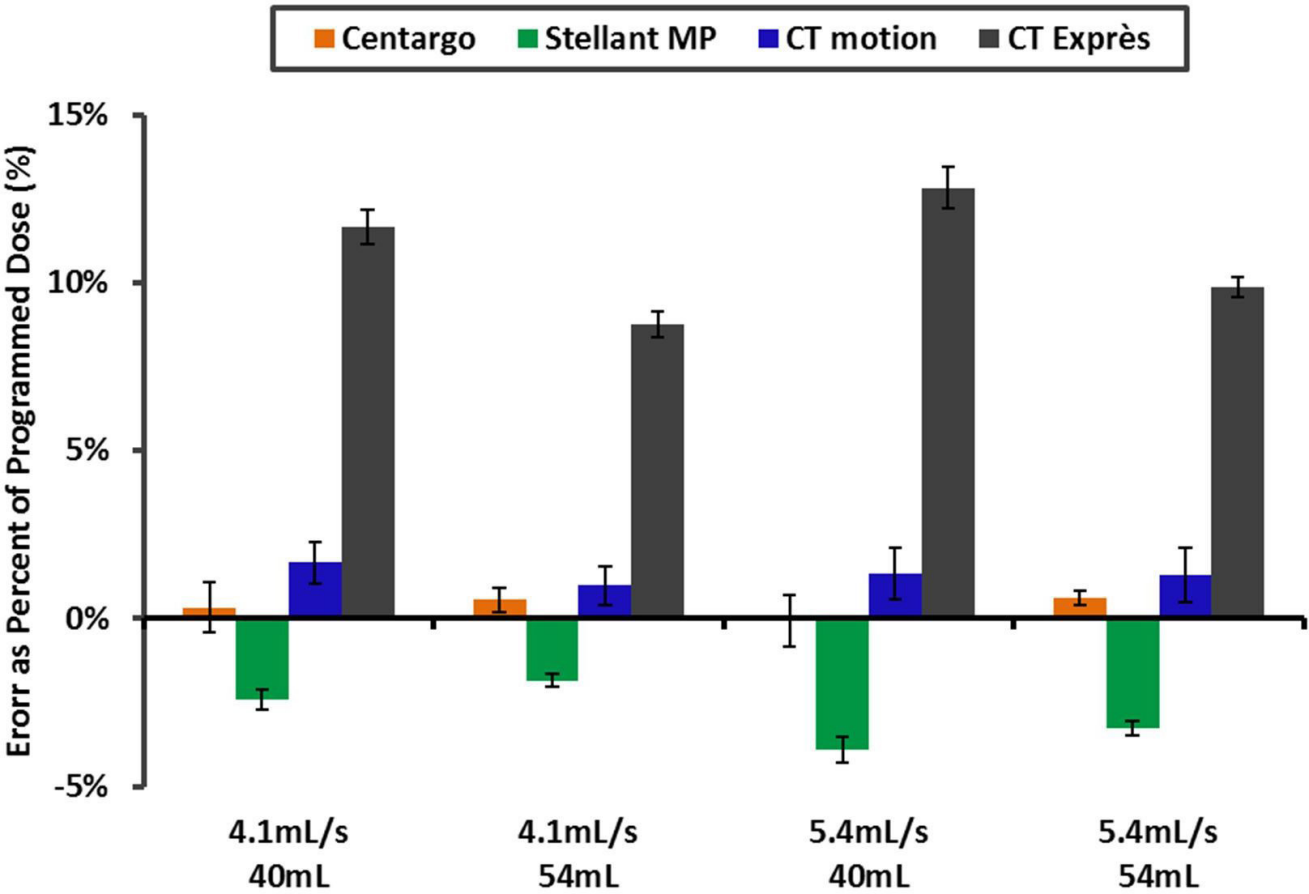
Contrast dose volume error with Ultravist 370 *﻿*vs injection system: this graph represents the results of the volume accuracy assessment for all injection systems considered. Results were obtained using a 20G catheter, and demonstrate a substantial over delivery of contrast media by CT Exprès compared to the other injection systems.

When reviewing the enhancement data, it was noted that trends in injection system performance were consistent from the PA to the AA. The exception was CT Exprès, which exhibited improved performance in the AA. To investigate this observation, volume accuracy performance was conducted on all systems. As shown in Figure 7, CT Exprès delivered 8–14% more contrast than the programmed volume. The operations manual for CT Exprès acknowledges the risk for inaccurate dosing when the catheter gauge as selected on the system is incorrect, which was required in this study to program equivalent IDRs on all systems. Conversely, all other injection systems did not exceed the programmed volume by more than 2.5%. The unintended increase in delivered iodine load explains improved performance of CT Exprès in the AA (Figure 4b and d) relative to the trends observed in the PA (Figure 4a and c).

## Limitations

With the use of the phantom instead of animal or human studies, only first-pass imaging can be performed. Therefore, these results cannot be directly translated to steady-state or parenchymal imaging. An additional limitation of the phantom is the relative size and internal blood volume, which roughly correspond to a 40 kg human. As such, the enhancement levels are artificially higher than one would expect for an average-sized patient. Finally, all CM were tested at room temperature, though warming may be performed clinically. This effect of temperature on CM viscosity is well established.^5^ Although testing was not completed at 37°C, the range of official viscosities in this study (9.2–27.5 cP) includes that of higher concentration CM heated to body temperature (*e.g.* iomeprol 400 at 37°C is 12.6 cP).^15^

## Conclusion

Piston-based injection systems, Centargo and Stellant MP, in combination with low viscosity CM provide higher achievable IDRs. This translates to higher peak vascular enhancement than the tested peristaltic injection systems, CT motion and CT Exprès. With IDRs achievable on all tested injection systems, Centargo provides higher peak vascular enhancement due to improved bolus shape.
